# Phoretic Poecilochirus mites specialize on their burying beetle hosts

**DOI:** 10.1002/ece3.3591

**Published:** 2017-11-07

**Authors:** Volker Nehring, Josef K. Müller, Nadine Steinmetz

**Affiliations:** ^1^ Department for Animal Ecology and Evolutionary Biology Biology I Freiburg University Freiburg Germany

**Keywords:** burying beetles, coevolution, cryptic species, host specialization, mites, parasites

## Abstract

Recurring species interactions can cause species to adapt to each other. Specialization will increase the fitness of symbionts in the coevolved association but may reduce the flexibility of symbiont choice as it will often decrease fitness in interactions with other than the main symbiont species. We analyzed the fitness interactions between a complex of two cryptic mite species and their sympatric burying beetle hosts in a European population. Poecilochirus mites (Mesostigmata, Parasitidae) are phoretic on burying beetles and reproduce alongside beetles, while these care for their offspring at vertebrate carcasses. While *Poecilochirus carabi* is typically found on *Nicrophorus vespilloides* beetles, *P. necrophori* is associated with *N. vespillo*. It has long been known that the mites discriminate between the two beetle species, but the fitness consequences of this choice remained unknown. We experimentally associated both mite species with both beetle species and found that mite fitness suffered when mites reproduced alongside a nonpreferred host. In turn, there is evidence that one of the beetle species is better able to cope with the mite species they are typically associated with. The overall fitness effect of mites on beetles was negative in our laboratory experiments. The *Poecilochirus* mites studied here are thus specialized competitors or parasites of burying beetles.

## 1 INTRODUCTION

Species in symbioses will often specialize on their main symbionts to the extent that fitness is reduced when associated with other species than the main symbiont. In these cases, there is a trade‐off between the degree of specialization on the main host and the fitness with alternative hosts, so that adaptations to traits specific to the main host are maladaptations to nonhosts (Lajeunesse & Forbes, 2002; Lively, 1999; Nosil & Harmon, 2009; Weissing, Edelaar, & van Doorn, 2011).

**Figure 1:**
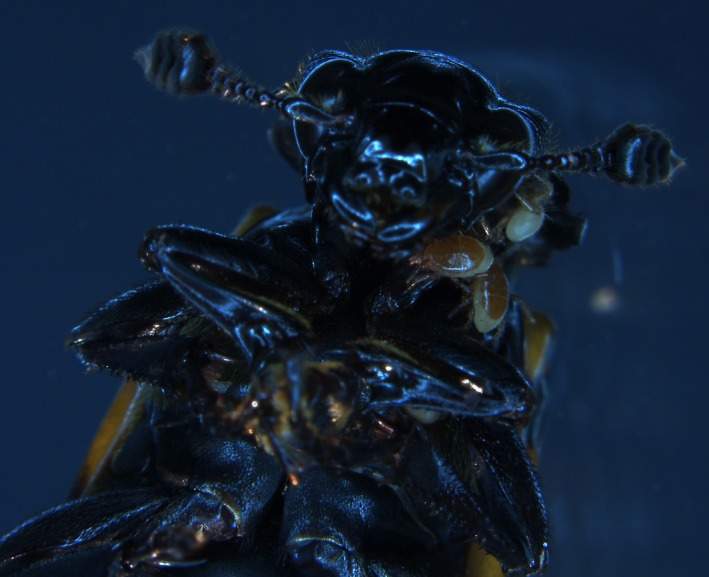
Deutonymphs of the mite *Poecilochirus carabi* use burying beetles for transport, here on the ventral side of their preferred host species, *Nicrophorus vespilloides*

An interesting symbiotic case is the association of phoretic *Poecilochirus carabi* mites and their *Nicrophorus* burying beetle hosts (Figure 1). The mites are very abundant and conspicuous, found on many species of burying beetles. A close inspection revealed that the mites comprise a species complex, with at least two different mite species found on different beetle species in both North American and European populations (described as *P. carabi* and *P. necrophori* for the European populations; Wilson, 1982; Müller & Schwarz, 1990; Brown & Wilson, 1992; Baker & Schwarz, 1997). Each mite species recognizes and prefers a different beetle species, and it is unknown whether the European and American mites are the same species. *Poecilochirus* mites are phoretic on the beetles and use beetles for transport to vertebrate carcasses (Eggert & Müller, 1997; Schwarz & Koulianos, 1998; Scott, 1998). While beetles bury and preserve the carcass and provide parental care to their offspring, the mites reproduce as well and mite offspring mount the parental beetles before these depart (Schwarz & Koulianos, 1998; Schwarz & Müller, 1992). In fact, mite development time seems to be adapted to the preferred beetle species' duration of parental care (Brown & Wilson, 1992; Schwarz & Müller, 1992; Schwarz, Starrach, & Koulianos, 1998). In North American *Poecilochirus* populations, there is evidence for fitness effects of mite host choice: Mites that choose *Nicrophorus tomentosus* beetles fare better on *N. tomentosus* than on *N. orbicollis* (Brown & Wilson, 1992). The fitness of mites choosing *N. orbicollis*, on the other hand, does not depend on the *Nicrophorus* species they are associated with.

The fitness effects that *Poecilochirus* mites have on their burying beetle hosts have been measured with varying results. *Poecilochirus* mites feed on the carcass, thereby compete with the beetles, and also eat beetle eggs and larvae (Beninger, 1993; De et al., 2015; De Gasperin & Kilner, 2015). Mites at a carcass also reduce the lifespan of brood‐caring *N. vespilloides* males, but not of females (De Gasperin & Kilner, 2015). On the other hand, *Poecilochirus* mites predate on nematodes and fly eggs, both competing with beetles for the resource, so that the beetles may benefit in some cases from mites removing competitors (Springett, 1968). At abnormally high densities, which can occur when a beetle reproduces multiple times and its mite population grows exponentially, North American *Poecilochirus* mites seem to harm their beetle hosts (Wilson & Knollenberg, 1987). However, single carcasses are typically visited by tens of burying beetles, which then directly fight for the resource. Losing beetles then often choose to reproduce as satellites along the dominant beetles instead of searching for a new carcass (Eggert & Müller, 1997; Müller, Braunisch, Hwang, & Eggert, 2006). It is thus unlikely that many individuals could breed four times or more in the field, which would be needed for the negative effects to manifest (Wilson & Knollenberg, 1987). Previous studies were conducted with all mite species that were present on the beetles (including uropodids, macrochelids, and histiostomatids) or without differentiating between different species of the *Poecilochirus carabi* complex, so that the precise interactions between the different mite and beetle species might have been obscured (De Gasperin et al., 2015; Wilson & Knollenberg, 1987).

We tested whether the European mites of the *Poecilochirus carabi* species complex, *P. carabi* and *P. necrophori*, affect their hosts' (*N. vespilloides* and *N. vespillo*, respectively) fitness. We experimentally infected pairs of both beetle species with both mite species separately under standardized laboratory conditions (no other competitors, fixed mite number and carcass weight). We hypothesized that beetle fitness should be reduced by the presence of mites. We also tested whether mites and beetles are adapted to each other. We predicted that each mite species' fitness would be higher with their respective preferred beetle species and that in turn, each beetle species coped better with the mites it is typically associated with. In addition, we also conducted a more sensitive analysis for a possible quantitative correlation of mite and beetle fitness.

## 2 METHODS

We set up pairs of beetles for reproduction on mouse carcasses in a device that allowed us to record when and with how many mites the beetles would leave the carcass. We used *N. vespillo* and *N. vespilloides* beetles that we infected with either *P. carabi*,* P. necrophori*, or no mites. We measured beetle fitness by counting and weighing beetle pupae and mite fitness by counting mite offspring.

### 2.1 Experimental animals

Beetles were direct offspring of individuals caught in the Mooswald forest close to Freiburg. The field‐caught beetles were bred under standardized conditions chosen to yield parasite‐free offspring of maximum size with little variance between offspring: Circa 12 larvae are reared by nematode‐free foster parents on a 20‐g mouse carcass (see Eggert et al., 1998; for details). *Nicrophorus vespillo* beetles were larger than *N. vespilloides* in both sexes (factor species *p* < .001, see Table S1 for details), and male beetles were slightly larger than female beetles in both species (factor sex *p* < .05, interaction sex*species *p* = .62; median pronotum size of *N. vespilloides* males 5.7 mm,/interquartile range 5.6–6.0 mm, females 5.6/5.4–5.7 mm; Nvo males 6.1/5.8–6.5 mm, females 6.0/5.4–6.3 mm). In the field, beetles vary greatly in size. While the species difference is robust and typically also apparent in field‐caught beetles, the slight difference between the sexes in our experimental beetles (0.1 mm) may be evident only under standardized laboratory conditions and not be important in the field.

Mites were deutonymphs from laboratory lines established with mites collected from their host beetles in the Mooswald forest a few weeks prior to the experiments. We discriminated *P. carabi* from *P. necrophori* by the beetles they were collected with and by their behavior in choice experiments (as was previously done by Baker & Schwarz, 1997; Müller & Schwarz, 1990): Mites collected with *N. vespilloides* were placed into an arena containing one beetle of each species. After 10 min, mites were removed from the beetles and those that chose *N. vespilloides* subsequently placed into a second arena offering the same beetle choice again. Only those mite individuals that chose *N. vespilloides* again were bred and used as *P. carabi* for the experiments. *Poecilochirus necrophori* was collected with *N. vespillo* and preferred this species twice. To simplify, we will indicate the mites' association with a preferred beetle host by referring to *P. carabi* with “Pvs” and to *P. necrophori* with “Pvo” throughout the method and result sections, according to their behavioral preference for *N. vespilloides* (“Nvs”) and *N. vespillo* (“Nvo”; Müller & Schwarz, 1990). The mites were bred without beetles on pieces of liver in peat‐filled boxes (Nehring & Müller, 2009; see also below, methods for mite reproduction time). All animal housing and breeding took place at 20°C, and all experiments were conducted at the same temperature.

### 2.2 Measuring fitness effects of the mite–beetle interaction

Pairs of beetle males and females were placed together in boxes (10 × 10 × 5 cm, lined with filter paper) to allow mating before the beginning of the experiment. After 1 day, a 10 g mouse carcass and for some treatments ten mite deutonymphs of one species were added to the box. We set up a total of 40 *N. vespilloides* pairs (*n* = 13 pairs without mites, *n* = 13 with Pvo mites, *n* = 14 with Pvs mites) and 41 *N. vespillo* pairs (*n* = 11 without mites, *n* = 15 Pvo, *n* = 15 Pvs). As soon as both beetles were manipulating the carcass, the carcasses with beetles and mites were gently moved to peat‐filled buckets (18 cm diameter, 16 cm height) with plexiglass disks as lids. After the mouse was buried, we replaced the plexiglass disk with an inverted bucket. After 2 days, an exit with a trap was added to the lid‐bucket so that beetles walking on the soil surface could exit the buckets and become trapped (details as in Müller, Eggert, & Dressel, 1990). We anesthetized all beetles caught in the trap and their mites with CO_2_ and counted mite deutonymphs.

We opened all *N. vespilloides* buckets approximately 20 days after the last beetle left the carcass and *N. vespillo* buckets after approximately 25 days. We anticipated that by this time all offspring would have pupated but no adults would have hatched. We removed and weighed beetle pupae individually and counted mites on pupae and those that remained in the soil. We chose to weigh pupae rather than larvae because the latter's weight varies until pupation, and pupation is not always successful. In contrast, pupal weight is relatively stable, which should reduce experimental error. In some cases, however, offspring beetles had already hatched earlier than expected so that we could not weigh them in the pupal stage. However, in a preliminary experiment where we measured the same *N. vespilloides* individuals as pupae and later again just after they hatched as adults, we found that pupal weight can be predicted from beetle mass (linear regression: intercept 50.4 mg (SE 20.4 mg), slope 0.94 (SE 0.11), *n* = 21, *p* < .001; *r* ^*2*^ **= .78). We thus calculated the expected pupal mass for individuals where we could only weigh beetles as mass(pupa) = mass(larva) * 0.94 + 50.4 mg. We also used the formula to predict the weight of some *N. vespillo* pupae. Because we cannot exclude that doing so might introduce noise or systematic error into the *N. vespillo* weight data, we conducted all potentially affected analyses a second time with a dataset from which we removed all *N. vespillo* replicates where some of the offspring had already hatched. The qualitative results (significance, approximate effect sizes) were not changed by removing these replicates, with the exception of one analysis where one trend became significant when the replicates were removed (see below).

### 2.3 Data analysis

We use total beetle brood mass, the sum of all individual pupal weights, as a proxy for host fitness because it draws an exact picture of how well the beetles translate their resource into offspring. We also analyze beetle offspring number; however, this measure cannot be compared between beetle species as adult size is species‐specific (see results). It also does not take into account that large offspring have a clear fitness advantage when in direct competition for resources, a situation that is quite common for burying beetles (Müller et al., 2006; Otronen, 1988).

Some beetle broods failed, and we used the likelihood of failed reproduction as a first fitness estimate to analyze potential harmful effects of mites on beetles. We did not only categorize broods with zero offspring as failed, but also three broods that yielded only very few offspring with low total mass of only 25%–34% of the average and 49%–65% of the minimum of all other replicates (933 mg, 966 mg, and 1251 mg), because of the large gap to the rest of the dataset. Similarly, we considered mite reproduction as failed if we did not recover more than the ten mites that we had initially added to the experiment, because these mites might have been the same individuals that entered the experiments (four replicates with *N. vespillo* beetles).

We analyzed the data with general linear models with Gaussian (for mass data), Poisson (offspring number), or binomial (brood failure) error families (R Development Core Team 2016). We started with the most complex models, including all reasonable explanatory factors and covariates (typically mite species and beetle species, depending on the question also beetle sex or mite offspring number; details of full models in Table S1) as well as all possible interactions. We reduced the models by stepwise removing terms until all remaining terms added significant explanatory value (based on Akaike information criterion and *p*‐values from *F* test (Gaussian models) or log‐likelihood test for sequentially dropped terms; details in Table S1).

### 2.4 Mite reproduction time

To estimate how much time the mites need for reproduction, we let mites reproduce separately from beetles in 10 × 10 × 5 cm boxes filled with a 2‐cm layer of peat. Ten mite deutonymphs of a species were provided with a ca. 0.5‐g piece of cattle liver placed on a Petri dish. We checked boxes for mites daily and scraped the liver surface to remove fungi and microbes that might prevent mites from feeding. Emerging deutonymphs of the next generation were collected using soft forceps or live beetles of the preferred host species. We placed the beetles into the box for two to three min and afterward anesthetized beetles and mites to count and remove the mites. We repeated this process until the beetles would not collect any more mites, and we did not see any remaining deutonymphs upon visual inspection.

## 3 RESULTS

### 3.1 Length of beetle parental care and mite development

The day beetles left their brood and the carcass and became trapped mostly depended on beetle species and sex. Males of both species left earlier than females (maximum likelihood (ML) test *p* < .001 for both species). *N. vespilloides* females (median/interquartile range 11/10–12.25 days) left much earlier than *N. vespillo* females (16/15–17.5 days; ML *p* < .001), but the difference between *N. vespillo* males (7/4.5–8 days) and *N. vespilloides* males (8/7–9 days; ML *p* < .01) was smaller (interaction sex*species *p* < .001, see Table S1 for details). There may be a slight effect of mite species on the day beetles left, with beetles from pairs infected with *P. necrophori* (Pvo) mites leaving less than a day earlier than beetles without mites (effect size partial η = 0.06; *p* = .058). There was no interaction of mite species with beetle sex or beetle species (all *p* > .4, see Table S1 for details). The difference in departure between males and females only depended on the beetle species (*p* < .001, see above), but was not influenced by differences in body size between male and female (*p* = .35), by the mites (*p* = .72) or any interaction (all *p* > .12).

When bred without beetles, the mite species differed in how long it took the next generation to become ready for dispersal. The first *P. carabi* (Pvs) deutonymphs (median/interquartile range 8/7–8 days) emerged earlier than the first Pvo deutonymphs (10/9–11 days, *p* < .01, *n* = 20 per species).

### 3.2 Overall beetle brood success

Some beetle broods failed. Broods with Pvs mites were more likely to fail than those with Pvo mites and without mites (*p* < .01; Pvs 37%, Pvo 4%, no mites 12%), and *N. vespilloides* pairs failed more often than *N. vespillo* pairs (*p* < .05; *N. vespilloides* 28%; *N. vespillo* 9%). There was no interaction between mite and beetle species affecting the likelihood of beetle failure (*p* = .43). In successful broods, *N. vespilloides* pairs (median 17 offspring) produced more offspring than *N. vespillo* pairs (13, *p* < .001). The total number of offspring was not influenced by the mite species (*p* = .25, interaction mite x beetle species *p* = .98).

**Figure 2:**
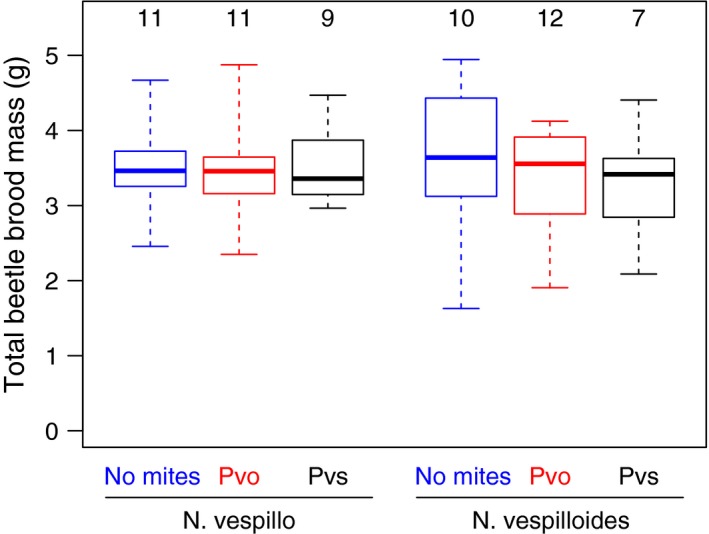
Total brood mass was comparable between *N. vespilloides* and *N. vespillo* beetles and was not influenced by mite presence or species (no mites, blue; Pvo: *P. necrophori*, red; Pvs: *P. carabi*, black). Boxplots depict median (thick line), interquartile range (box), minimum and maximum. Numbers above boxes are sample sizes

In a comparison among the six treatment groups (3 mite regimes × 2 beetle species), total beetle brood mass was not influenced by beetle species (Figure 2, *p* = .78), mite presence or species (*p* = .62), or their interaction (*p* = .96). This means that both beetle species use carcasses equally efficient and translate ca. 36% of the carcass mass into offspring mass. The two beetle species produce offspring of different size (average pupal mass *N. vespillo* 276 mg vs. *N. vespilloides* 211 mg), which parents control by managing the number of offspring they raise (Bartlett, 1987). In our dataset, offspring number had a negative effect on offspring size (Fig. S1), which is in line with earlier observations and likely caused by competition among offspring (Smiseth et al., 2007). Interestingly, the effect is less pronounced in *N. vespilloides* (interaction offspring number x beetle species *p* < .001), where offspring number was more variable. The difference between the species could partly be caused by different putative thresholds for minimal larval sizes (Monteith et al., 2012): Adding an additional larva to a brood would be more costly for the siblings in *N. vespillo* because more resources would be used (minimal larval size is bigger), and costs are distributed over fewer siblings in *N. vespillo* than in *N. vespilloides*, causing a steeper slope for *N. vespillo*.

### 3.3 Mite fitness

**Figure 3:**
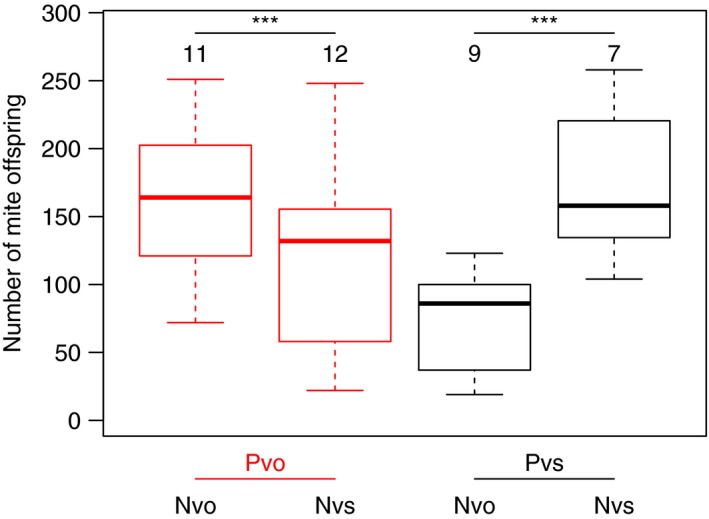
Mite fitness depends on the combination of mite fitness and beetle fitness. Each mite species has a higher fitness with the beetles it is typically found on (*P. necrophori* (Pvo, red) with *N. vespillo* (Nvo); *P. carabi* (Pvs, black) with *N. vespilloides* (Nvs)). Numbers are sample sizes; ***p < .001 in a maximum likelihood test

Mites never failed to reproduce along with successful *N. vespilloides* beetles, but in 16% of the successful *N. vespillo* broods, mite reproduction failed (glm *p* < .05). Mite species (*p* = .93) or the interaction with beetle species (*p* > .99) did not affect the likelihood of mite failure. The fitness of successful mites, however, as measured in total offspring number, strongly depended on the combination of mite and beetle species (Figure 3, interaction mite species × beetle species *p* < .001). While the fitness of each mite species with its preferred beetle species was equally high, it dropped when paired with the nonpreferred beetle species by approximately 20% (Pvo) and 55% (Pvs). The effect is amplified when taking only mite offspring into account that ended up on the parental beetles that left the carcasses: Then, the fitness of Pvo, the mite species with the longer development time, dropped by 35% when reproducing along with *N. vespilloides*, the nonpreferred beetle host, which has a brood care duration time that is 30% shorter than that of the preferred host *N. vespillo* (see above).

### 3.4 Interaction of mite and beetle fitness

**Figure 4:**
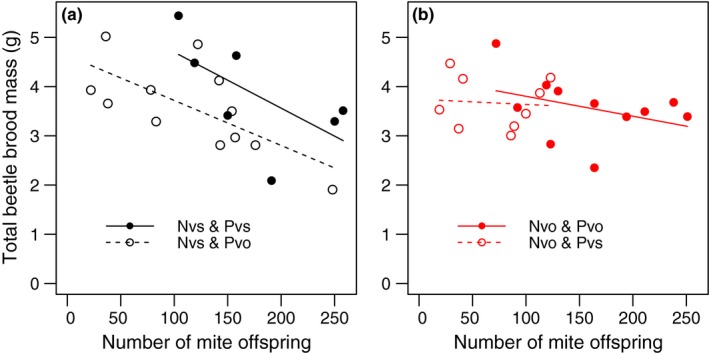
Beetle fitness (measured as total brood mass) and mite fitness (offspring per 10 individuals) are overall negatively correlated, indicating that the mites are parasites. *P. carabi* (Pvs, black) prefers *N. vespilloides* beetles (Nvs, (a)), and *P. necrophori* (Pvo, red) prefers *N. vespillo* (Nvo, (b)). Beetle fitness is also higher when beetles are paired with their specialized mites (solid regression lines of preferred combinations are above those of dashed alternatives)

We also tested for an association of mite and beetle fitness in the successful beetle and mite breeding attempts (Figure 4). We found a negative correlation of beetle and mite fitness (*p* < .01). This effect seemed weaker in breeding attempts with *N. vespillo* beetles (Figure 4b; interaction mite fitness x beetle species *p* < .05), where mite offspring numbers varied much between Pvs and Pvo mites so that a formal comparison of the effects is difficult for *N. vespillo* (see also Figure 3). The interaction between beetle and mite species came close to being significant (*p* = .06) and explained a substantial amount of the total variation in beetle fitness (partial η^2^ = 0.10, see also Table S1). When we excluded *N. vespillo* replicates in which some pupae had already hatched because our prediction of pupal size may be less accurate, the interaction between beetle and mite species became marginally significant (*p* = .046, partial η^2^ = 0.16). This means that taking the effect the number of mite offspring into account, beetle fitness was approximately 30% (*N. vespilloides*, Wald's *p* < .05) and 4.5% (*N. vespillo p* = .66) lower when beetles were paired with the mite species they do not typically carry. In the previous analysis ignoring the effect of mite fitness (Figure 2), the interaction of mite and beetle species on beetle fitness was likely obscured by the fact that mite fitness was lower when mites were associated with the nonpreferred beetle species. In these cases, fewer mite offspring were produced (Figure 3) so that beetles suffered less from large mite numbers than when associated with their typical mite species. In other words, beetles parasitized by nonspecialized mites were not able to benefit from the numerically lower parasite pressure in these breeding attempts. The effect of mite offspring number was similar to the number of beetle offspring as it was on beetle brood mass, with negative effects on *N. vespilloides* beetles but only small effects on *N. vespillo* (interaction mite fitness x beetle species *p* < .01). However, the mite x beetle species interaction was not evident in this analysis (*p* = .35), likely covered by the strong inherent difference in offspring number between the beetle species (*p* < .001).

## 4 DISCUSSION

We analyzed the effects of symbiotic burying beetles and *Poecilochirus* mites on each other's fitness, with a particular focus on a specialization of mite and beetle species on one of the two sympatric symbiont species. The fitness of beetles with and without mites did not differ, which at first glance suggests that mites are beetle commensalists with no significant fitness effects. However, on closer inspection, it became clear that mites impede beetle fitness when mites are very successful. There was a negative effect of mite fitness on beetle fitness, suggesting mites are parasites or competitors of beetles. Mites of both species are more successful when reproducing along their preferred beetle species than another beetle (Figure 3), and *N. vespilloides* beetles seemed to cope better with their usual *P. carabi* mites (Figure 4).

### 4.1 Mite specialization

Mites are clearly specialized on their preferred host species. *Poecilochirus carabi* (Pvs), the species preferring *N. vespilloides* over *N. vespillo* (Müller & Schwarz, 1990), had a higher fitness with *N. vespilloides* beetles. *Poecilochirus necrophori* (Pvo), in contrast, prefers *N. vespillo* and had a higher fitness with its preferred host species. These fitness effects of host choice partly derive from mite generation time being adapted to the brood care duration of the preferred beetle host (Brown & Wilson, 1992; Müller & Schwarz, 1990). Reproduction of *P. necrophori* took about 10 days, and the last *N. vespillo* parent, typically the female, stays until day 16 under the conditions that applied in our experiments (20°C, 10‐g mouse carcass). The last *N. vespilloides* parent left on average 5 days earlier, which led to at least some *P. necrophori* mites missing this beetle in most replicates (up to 50% in single replicates) when matched with the wrong host species. The carcasses are well hidden by beetles and would be of little value to other arthropods when the beetle brood is fully developed, because they typically consume them entirely (in the present study, we found remnants of the carcass other than hairs in only ca. 6% of the successful breeding attempts). Mites that miss the parental beetles are unlikely to be able to disperse before the beetle offspring hatch a month later (Müller & Schwarz, 1990; Schwarz & Koulianos, 1998). As is obvious from our results, the generation time cannot explain all fitness reduction on host mismatch because all *P. carabi* mites should be quick enough to reach the parental beetles of any host species but still suffer fitness costs from associating with *N. vespillo*, and also in *P. necrophori* the mismatched timing explained less than half of the total fitness reduction.

The observation that the European *Poecilochirus* mites used in our experiments specialize on a host beetle species is in line with the fact that one American *Poecilochirus* species is specialized on *N. tomentosus* (Brown & Wilson, 1992), although both of the European and only one of the American species suffered fitness costs from mismatched host beetles. We have little understanding of what species‐specific beetle traits the mites may have adapted to, other than the duration of brood care. We know that mite females eat beetle eggs when they have the chance (Beninger, 1993). The eggs may be a superior diet for mites that increase egg production rate or female longevity, leading to more reproductive output per individual. Both beetle species lay their eggs away from the carcass, which might be a strategy to prevent negative effects of symbionts such as mites, bacteria, or fungi. *Nicrophorus vespillo* lays the eggs along a single gallery (Pukowski, 1933), while *N. vespilloides* females scatter their eggs along hard surfaces in the soil (JKM, VN, pers. obs.). One might thus speculate that adopting a host species‐specific “egg hunt” strategy may increase mite fitness when reproducing alongside the preferred host.

Other specific diet could be represented by nematodes: The two beetle species are suspected to carry different nematode species (Richter, 1990). However, the beetles in our experiments were nematode‐free so that any influence of nematode variation on our results is unlikely. Finally, the beetles manipulate the microbiota growing on the carcass (Arce et al., 2012; Cotter & Kilner, 2010; Duarte et al., 2017; Vogel et al., 2017). If microbiotic communities caused by different beetle species would differ, the two mite species may have evolved different strategies to deal with them. In any case, given how little mites of the *Poecilochirus carabi* species complex diverged morphologically and behaviorally, and considering the host specialization observed in our experiments, it is not unlikely that divergent host use between populations was a driving force behind *Poecilochirus* speciation (Magalhães et al., 2007; Nosil & Harmon, 2009).

### 4.2 Fitness interactions

It is surprising that no fitness effects are apparent from comparing beetle broods with and without mites. However, beetle fitness was generally very variable, and low beetle fitness was associated with high mite fitness. One potential reason might be that the mites may directly reduce beetle fitness, for example, by preying on beetle offspring. The beetles lay “backup eggs” and replacement clutches so that predation on a few beetle eggs would not reduce the overall brood size nor the brood mass (Bartlett & Ashworth, 1988; Müller, 1987; Müller, Eggert, & Furlkröger, 1990), but if mite offspring killed beetle larvae (De Gasperin & Kilner, 2015), this could explain at least some part of the negative effects successful mite reproduction has on beetle offspring number.

As killing of some beetle larvae would reduce competition among beetle larvae, thereby allowing individual larvae to grow bigger (Smiseth et al., 2007), mite predation can only explain reduced offspring numbers but not necessarily the reduction in total brood mass. Instead, mite offspring and beetle larvae may compete for resources, most likely the carrion food, as has been suggested by De Gasperin et al. (2015). Then, beetle fitness would suffer when mites produce many offspring. As the mites are horizontally transmitted, there is little inherent limitation on how virulent the mites can evolve to be (Ebert & Herre, 1996), because their fate is uncoupled from that of the beetle offspring.

Mite offspring number was variable, which might be due to the fact that we controlled for initial mite density, but not for mite sex ratio. Mite deutonymphs of both sexes are virtually indistinguishable. Consider that some beetle pairs started out with eight female and two male mites, and others with the reversed sex ratio. There is strong interference competition among male mites but not among female mites (Nehring & Müller, 2009) so that all females are likely to lay eggs. Thus, more offspring would be expected in the first scenario, and, through competition of mite offspring with beetle larvae, a larger fitness effect of mites on the beetles. Alternatively, mites and beetles may engage in a tug‐of‐war, and the outcome may differ depending on how good a specific mite phenotype is at exploiting the particular beetle phenotype under the given environmental conditions. If these phenotypes were heritable, fluctuating red queen dynamics (Brockhurst et al., 2014) could cause genetic variation in the decisive traits involved in the interaction.

The fitness correlation could in theory also be caused purely by beetle variation, affecting the mites only secondarily. If for reasons unrelated to the mites, beetle reproduction were sometimes suboptimal, fewer resulting beetle larvae might consume fewer resources. Then, more resources would be available for the mites, which consequently produce more offspring. Several lines of evidence make this scenario unlikely. First, beetle larval number and larval size are negatively correlated. If some beetle larvae die early on, the remaining larvae will grow bigger, using the same resources and reaching a similar total brood mass as would broods with more larvae (Smiseth et al., 2007). In our experiments, both beetle species translate around 35% of the carcass mass into brood mass, but differ in the number of larvae and the average mass per larva, so that individual larvae became smaller when there were more larvae (Fig. S1). It is thus unlikely that reducing larval number will leave more resources for the mites. Second, the fitness correlation between mites and beetles depends on the combination of species that interact. Both beetle fitness and mite fitness are higher in the same combinations, those typically found in the field (see also below). If lower beetle fitness would leave more resources for the mites, mite fitness should benefit particularly in those species combinations where beetle fitness is lower, that is, the nonpreferred combinations, which is the opposite of what we found. A direct or indirect negative effect of mites on beetles is thus a more parsimonious explanation for the fitness correlation we observed. However, for a conclusive answer to the question what causes the negative fitness correlation between mites and beetles, dedicated experiments would be necessary. In addition, under specific conditions in the field, mites may even benefit the beetles by, for example, keeping competitors (flies) at bay (as suggested by Springett, 1968). To test this was beyond the scope of this study and deserves attention in future experiments.

### 4.3 Beetle specialization

While mite specialization on a preferred beetle species is the strongest effect in our dataset, our results also suggest that there may be a specialization of the beetles on their “preferred” mites, the mites they are typically associated with in the field. There is much variation in beetle fitness, partly unexplained and partly explained by the number of mite offspring. We found in particular for *N. vespilloides* that beetle fitness is lower when paired with *P. necrophori* mites than with the typical *P. carabi* symbionts. This could mean that the beetles actually have adapted to cope with the mites they are typically encountering in the field. While our data do not show a similar specialization by *N. vespillo* beetles, it is possible that its effects are too weak to be evident in our small dataset and may be obscured by a suboptimal prediction of pupal mass in incidences where adults had already hatched before we could weigh the offspring. As is true for the specialization of mites on the beetles, we can only speculate about how the beetles may adapt to a specific mite species. It is unlikely that beetles somehow hinder mite reproduction early on, as mites produce more offspring in the preferred combinations. More likely, the beetles may dodge specific negative effects caused by their specific mite species and can thus not be explained purely by scramble competition between beetle larvae and mites for food. The effects need not necessarily be caused directly by the mites themselves, but may result from third parties. If the mites carried specific microbes, for example, beetle anal secretions may be specifically targeted at these (Cotter & Kilner, 2010; Duarte et al., 2017). In any case, the potential for beetle adaptations to mites deserves to be studied with specifically designed experiments.

### 4.4 Stronger specialization in *P. carabi*

It is interesting to note that *N. vespilloides* fitness and brood success were more sensitive to mite effects, and *N. vespilloides* was also more specifically adapted to their typical mites than *N. vespillo*. At the same time, *P. carabi* mites are more virulent (caused more beetle broods to fail and had a stronger correlation between mite and beetle fitness than *P. necrophori*) and suffer greater fitness loss on host mismatch than *P. necrophori*. This points at a greater specialization in the *N. vespilloides*—*P. carabi* association, with more or stronger reciprocal adaptations. A greater specialization is expected when associations are more stable, for example, when the parasites do not use any alternative hosts (Lajeunesse & Forbes, 2002). However, as far as we know, *P. carabi* mites can actually be found in three sympatric species of burying beetles, while *P. necrophori* is mainly restricted to *N. vespillo* (in another population, Schwarz et al., 1998). Perhaps the stronger specialization of *P. carabi* can be better explained by a qualitative than a quantitative view on host range. *N. vespilloides* is by far the most abundant burying beetle in the German populations studied so far, with *N. vespillo* being far behind, and all other species rather rare (Dressel, 1985; Schäfer, 2000; Schwarz & Koulianos, 2000). Under such circumstances, a close specialization on *N. vespilloides* would be profitable because this species is always available. *P. necrophori* has found an alternative niche by adapting to *N. vespillo*, but as this beetle is less common, *P. necrophori* is forced to still use *N. vespilloides* from time to time (Schwarz et al., 1998), which makes a close tracking of its main host's traits less profitable than it is for *P. carabi* and the abundant *N. vespilloides*.

## 5 CONCLUSIONS

*Poecilochirus carabi* and *P. necrophori* mites have specifically adapted to their preferred host beetle species, *N. vespilloides* and *N. vespillo*, respectively. The mites suffer fitness costs when associating with the wrong beetle species. Beetle and mite fitness are negatively correlated, possibly due to a tug‐of‐war fought on every resource separately, although direct experimental evidence for this hypothesis is still lacking. There is also evidence that one of the beetle species, *N. vespilloides*, has specifically adapted to the mite species they typically encounter, suggesting a history of coevolution with reciprocal adaptations by both symbionts, mites and beetles. The host specialization among multiple interacting host species and parasite species, with diffuse but measurable fitness effects, makes the *Poecilochirus*–*Nicrophorus* system a promising model to study the more subtle types of coevolution.

## DATA AVAILABILITY

The data are available from the Dryad Digital Repository: https://doi.org/10.5061/dryad.6c8v6.

## CONFLICT OF INTEREST

None declared.

## AUTHOR CONTRIBUTIONS

VN & JKM planned the experiments; VN & NS conducted the experiments; VN analyzed the data; and all authors contributed to writing the manuscript.
