# Supplementary material for: Phoretic Poecilochirus mites specialize on their burying beetle hosts
Source: Ecol Evol. 2017 Nov 7;7(24):10743–51. doi: 10.1002/ece3.3591 (PMC5743630; doi:10.1002/ece3.3591)
Supplement: Supplementary file 1 [file ECE3-7-10743-s001.pdf]

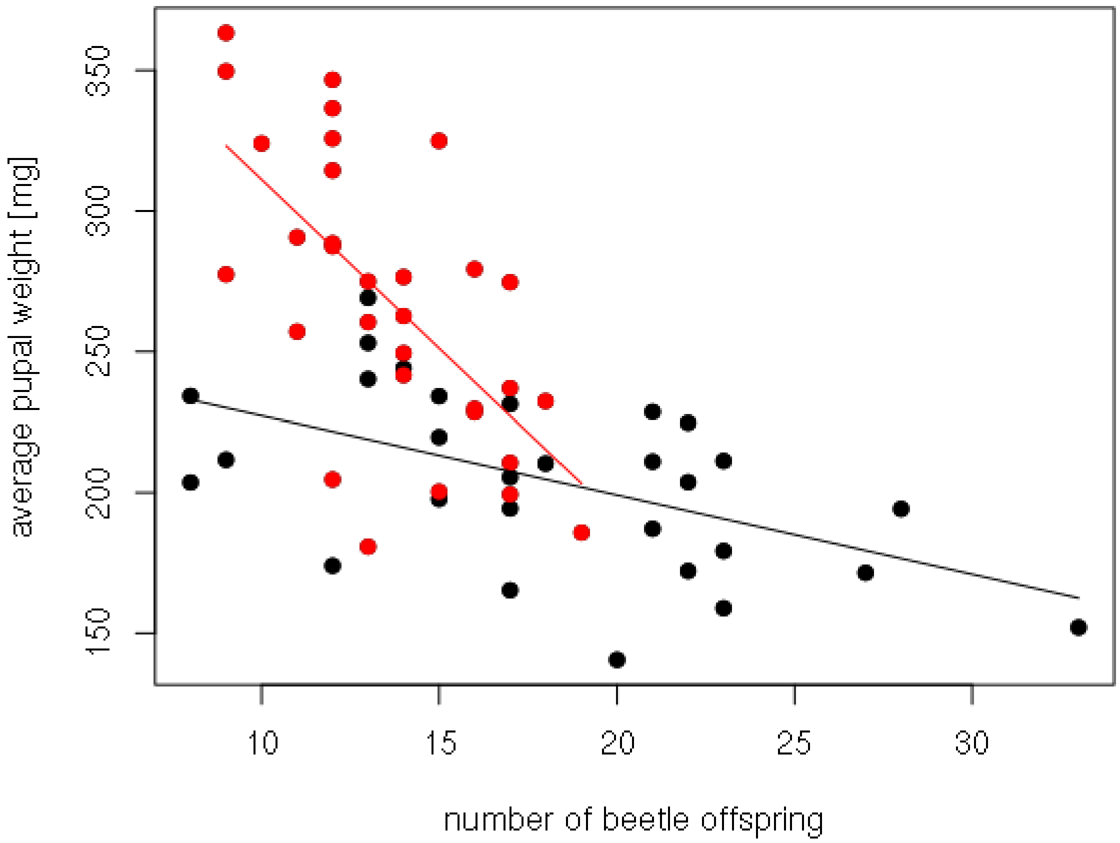

Supplementary Figure 1: The effect of offspring number on pupal weight is negative in *N. vespilloides* (black, $p < 0.01$) and *N. vespillo* (red, $p < 0.001$) but differs between the beetle species ($p < 0.001$, $r^2 = 0.61$).
