# Supplementary material for: Phoretic Poecilochirus mites specialize on their burying beetle hosts
Source: Ecol Evol. 2017 Nov 7;7(24):10743–51. doi: 10.1002/ece3.3591 (PMC5743630; doi:10.1002/ece3.3591)
Supplement: Supplementary file 2 [file ECE3-7-10743-s002.pdf]

Supplementary material 1: Statistical analyses for all generalized linear models mentioned in the manuscript. Each analysis started with the most complex model possible (maximal model) and was then reduced by factors or interactions that were not contributing to explaining the variance (based on comparisons of the Akaike Information Criterion, AIC and log ratio tests). These factors and their test statistics are listed first, followed by the factors contributing to the final model and their test statistics and effect sizes (*partial* η^2).

Size of parental beetles

Factor	*AIC without factor*	*log ratio*	*p*	
maximal model	170.47			
- species x sex	168.72		0.63	
final model:	*Deviance*	*df*	*p*	*partial* η^2
sex	1.04	1	0.04	0.00
species	5.32	1	< 0.001	0.01
Residuals	26.81	117		

Day beetles left the carcass

Factor	*AIC without factor*	*log ratio*	*p*	
maximal model	591.8			
- beetle species x mite species x beetle sex	588.99	1.19	0.55	
- beetle species x mite species	586.2	1.2	0.55	
- beetle sex x mite species	583.8	1.59	0.45	
final model:	*Deviance*	*df*	*p*	*partial* η^2
species	4.74	1	0.029	0.05
sex	122.85	1	< 0.001	0.58
mite species	5.71	2	0.0576	0.06
beetle species x beetle sex	24.53	1	< 0.001	0.22
Residuals	87.89	112		

time between male and female beetle left

Factor	*AIC without factor*	*F*	*p*	
maximal model	324.58			
- size difference x beetle species x mite species	324.83	1.54	0.23	
- size difference x beetle species	323.8	1.01	0.32	
final model:	*Deviance*	*df*	*p*	*partial* η^2
size difference	11.56	1	0.35	0.02
beetle species	640.17	1	< 0.001	0.50
mite species	8.28	2	0.73	0.01
size difference x beetle species	43.46	2	0.2	0.06
beetle species x mite species	55.62	2	0.13	0.08
Residuals	639.33	49		

Beetle reproduction successful?

Factor	*AIC without factor*	*log ratio*	*p*	
maximal model	66.55			
-mite species x beetle species	64.24	1.69	0.43	
final model:	*Deviance*	*df*	*p*	*partial η^2*
mite species	10.21	2	0.006	0.15
beetle species	5.75	1	0.16	0.09
Residuals	56.24	71		

Number of beetle offspring in successful broods

Factor	*AIC without factor*	*log ratio*	*p*	
maximal model	355			
-beetle species x mite species	351	0.04	0.99	
-mite species	350	2.73	0.26	
final model:	*Deviance*	*df*	*p*	*partial η^2*
beetle species	20	1	< 0.001	0.22
residuals	72.5	58		

Beetle brood weight in successful broods

Factor	*AIC without factor*	*F*	*p*	
maximal model	985			
- beetle species x mite species	981	0.05	0.96	
- mite species	978	0.47	0.63	
- beetle species	976	0.08	0.78	
final model:	*Deviance*	*df*	*p*	*partial η^2*
residuals	38147168	59		

Tradeoff between beetle offspring number and average pupal weight

Factor	*AIC without factor*	*F*	*p*	
maximal model	439			
- beetle offspring x beetle species x mite treatmen	437	0.7	0.5	
-beetle species x mite treatment	433	0.09	0.91	
- beetle offspring x mite treatment	430	0.4	0.67	
- mite treatment	428	0.77	0.47	
final model:	*Deviance*	*df*	*p*	*partial η^2*
beetle offspring	63877	1	< 0.001	0.49
beetle species	22302	1	< 0.001	0.25
beetle offspring x beetle species	15493	1	< 0.001	0.19
	65270	56		

Mite reproduction successful?

Factor	*AIC without factor*	*log ratio*	*p*	
maximal model	30			
- beetle species x mite species	28	3.98E-011	> 0.99	
- mite species	26	0.008	0.93	
final model:	*Deviance*	*df*	*p*	*partial η^2*
beetle species	4.82	1	0.28	0.18
residuals	21.98	42		

Mite offspring number in successful broods

Factor	*AIC without factor*	*log ratio*	*p*	
maximal model	1259			
final model:	*Deviance*	*df*	*p*	*partial η^2*
beetle species	26.2	1	< 0.001	0.03
mite species	27	1	< 0.001	0.03
beetle species x mite species	426.4	1	< 0.001	0.30
residuals	996.7			

Beetle brood weight depending on mite offspring number

Factor	*AIC without factor*	*F*	*p*	
maximal model	630			
- mite number x mite species x beetle species	628	0.3	0.57	
- mite number x mite species	626	0.004	0.95	
final model:	*Deviance*	*df*	*p*	*partial η^2*
mite number	3806923	1	0.007	0.20
mite species	66213	1	0.704	0.00
beetle species	222096	1	0.488	0.01
mite number x beetle species	2042578	1	0.041	0.12
mite species x beetle species	1653602	1	0.065	0.10
residuals	14918191	33		

Beetle offspring number depending on mite offspring number

Factor	*AIC without factor*	*log ratio*	*p*	
maximal model	225			
- mite number x mite species x beetle species	223	0.0001	0.99	
- mite number x mite species	221	0.14	0.7	
- mite species x beetle species	220	0.85	0.36	
final model:	*Deviance*	*df*	*p*	*partial η^2*
mite number	3.9	1	0.05	0.11
mite species	0.4	1	0.53	0.01
beetle species	16.3	1	< 0.001	0.35
mite number x beetle species	8.7	1	0.003	0.22
residuals	30.53			
